# *Moniezia benedeni* infection enhances neuromedin U (NMU) expression in sheep (Ovis aries) small intestine

**DOI:** 10.1186/s12917-022-03243-2

**Published:** 2022-04-19

**Authors:** Wan-Ling Yao, Li-Ping Liu, Yan-Qiao Wen, Bao-Shan Wang, Jia-Qi Dong, Wan-Hong He, Xi-Ping Fan, Wen-Hui Wang, Wang-Dong Zhang

**Affiliations:** College of Veterinary Medicine, Gansu Agricultural University, Lanzhou, 730070 Gansu China

**Keywords:** Sheep, Small intestine, *Moniezia benedeni* infection, NMU, Prokaryotic expression, Distribution

## Abstract

**Background:**

Neuromedin U (NMU) plays an important role in activating the group 2 innate lymphoid cells (ILC2s) and initiating the host’s anti-parasitic immune responses. It is aimed to explore the distribution characteristics of NMU in the sheep small intestine and the influence of *Moniezia benedeni* infection on them. In the present study, the pET-28a-NMU recombinant plasmids were constructed, and *Escherichia coli.* BL21 (DE3) were induced to express the recombinant protein. And then, the rabbit anti-sheep NMU polyclonal antibody was prepared and immunofluorescence staining was performed with it. The expression levels of NMU in the intestine of normal and *Moniezia benedeni-*infected sheep were detected by ELISA.

**Results:**

The results showed that the molecular weight of the obtained NMU recombinant protein was consistent with the expected molecular (13 kDa) and it was expressed in the form of inclusion body. The titer and specificity of obtained rabbit anti-sheep NMU polyclonal antibody were good. The results of immunofluorescence analysis showed that the nerve fibers which specifically expressed NMU mainly extended from the ganglion in the submucosal to *lamina propria* (LP) in the sheep small intestine, and the expression level was relatively high; especially on the nerve fibers of LP around the intestinal glands. The expression levels were gradually increased from the duodenum to the ileum, and the levels in the jejunum and ileum were significantly higher than that in the duodenum (*P* < 0.05). In addition, scattered NMU positive cells were distributed in the epithelium of the jejunal crypts. *Moniezia benedeni* infection increased the expression of NMU in each intestinal segment, especially in the jejunum and ileum there were significant increase (*P* < 0.05).

**Conclusions:**

It was suggested that *Moniezia benedeni* infection could be detected by the high expression of NMU in sheep enteric nervous, and which laid the foundation for further studies on whether NMU exerts anti-parasitic immunity by activating ILC2s. In addition, NMU was expressed in some intestinal gland epitheliums, which also provided a basis for studying its roles in regulation of the immune homeostasis. The present study laid the foundation for further revealing the molecular mechanism of sheep’s neural-immune interaction network perceiving the colacobiosis of parasites.

**Supplementary Information:**

The online version contains supplementary material available at 10.1186/s12917-022-03243-2.

## Background

In humans and animals, the intestine is the important place for digesting food and absorbing nutrients. Meanwhile, because it directly communicates with the external environment, the intestinal lumen is also always facing the threat of complex potential antigens such as food antigens [1, 2], toxins [3, 4], bacteria [5–7], viruses [8, 9], fungi [10–13] and parasites and their metabolites [14–16]. In recent years, studies have found that enteric neurons and the intestinal immune system could form a complex interactive regulatory network [17], which could accurately monitor and respond to the potential threats from intestinal lumen antigens through the highly coordinated response [18–20]. Therefore, the enteric neuroimmune network is the core of regulating intestinal homeostasis.

Neuromedin U (NMU), first isolated from pig spinal cord, is a neuropeptide with the ability to contract rat uterine smooth muscle [21–26]. Subsequently, it was isolated from various animals such as rats [27], guinea pigs [28], rabbits [29], dogs [30], avians [31, 32], frogs [33], etc. and its structure was highly conserved. Studies have shown that NMU plays multiple biological roles mainly by the binding of ligands to its specific receptors NMUR1 and NMUR2 [34, 35], such as participating in the regulation of smooth-muscle contraction [36], blood pressure and local blood flow [37, 38], intestinal ion transport, stress response [39, 40], feeding and energy homeostasis [35], cancer, gastric acid secretion, pronociception and feeding behaviors, etc. [41]. In particularly the latest researches have shown that cholinergic neurons could express NMU, and which co-localized with ILC2s in the mouse gastrointestinal tract. Meanwhile, the ILC2s could express NMU receptor 1 (NMUR1) selectively [42]. And in vitro experiments confirmed that ILC2s could be activated rapidly by NMU and proliferated in vitro, and then the type 2 cytokines IL-5, IL-9 and IL-13 were produced and secreted, which depended on the expression of NMUR1 and Gαq in ILC2s. On the contrary, the susceptibility of Nmur1^−/−^ mice to worm is higher than that of control mice, and gene-deficient mice and adaptive cell transfer experiments showed that NMU played an important role in inducing type 2 immune response with ILC2s as the core [43].

Parasites are the important pathogens of human and animals. They can parasitize in their hosts for a long time, some even accompany them for life if untreated. In fact, a state of long-term coexistence between hosts and the parasites is formed, which is because the parasites could successfully escape or inhibit the host's immune sensing or clearance [14]. Studying the molecular mechanism of the host’s perception on parasite infection, in-depth understanding the interaction between parasites and hosts, and further seeking the potential drugs for treatment of some human intestinal allergic diseases or immune system tumors (such as colon cancer, etc.) from the body of parasites or their metabolites have very important application value. The *Moniezia benedeni* belongs to the *Anoplopcephalidae* family and *Moniezia* genus. The body, whose back and abdomen are flat, is milky white and banded. The length is more than 2 m. The adult consists of the scolex, neck and strobila. It is a large parasite usually parasitizing in the sheep small intestine. This parasitic disease was first reported in the world in 1944. *Scheloribates.sp* is its main intermediate host. The disease is distributed worldwide and mostly endemic. *Moniezia benedeni* is one of the most important pathogens of sheep tapeworm disease, which mostly occurs in lambs aged 5 to 7 months. When the infection is severe, it could cause death. The adult sheep are also infected, but the infection symptoms of are mild. At present, there are many reports on the morphology, life history, insect somatic body genes, diagnosis and treatment of the disease, and epidemiological investigations of *Moniezia benedeni*. However, the researches about the perception mechanism and corresponding anti-parasitic immune response of the host in the *Moniezia benedeni* infection of sheep have been reported less, especially the impact on the expression of NMU in sheep small intestine.

In the present study, a rabbit anti-sheep NMU polyclonal antibody with good specificity was prepared by bioinformatics analysis of sheep NMU gene and construction of a prokaryotic expression system. On this basis, the distribution characteristics and expression levels of NMU in the small intestine of healthy and *Moniezia benedeni*-infected sheep were analyzed. Furtherly, we researched the impact of *Moniezia benedeni* infection on the NMU expression in sheep small intestine. The present study would lay the foundation for further revealing the molecular mechanism of mucosal immune network of sheep digestive tract perceiving the parasitism of parasites.

## Materials and methods

### Experimental design and sample preparation

Healthy (Normal group, *n* = 6) and *Moniezia benedeni*-infected sheep (Infected group, *n* = 6) were selected respectively. They were anaesthetised intravenously with sodium pentobarbital (20 mg/kg) and then exsanguinated to death. The abdomen of each sheep was cut open, and the whole small intestine were obtained removed from the pylorus of the abomasum to the ileocecal aperture. The intestinal contents were removed and the mucosal surface was thoroughly washed by saline. All the tissue samples of the duodenum, jejunum and ileum for ELISA and Western Blotting detection were collected in sterile tube and immediately put into liquid nitrogen, and then transferred to -80 ℃ refrigerator for further use. And the histological samples of them were fixed in a 4% neutral paraformaldehyde solution for more than 15 days.

### Synthesis of sheep NMU gene and construction of recombinant plasmid

The mRNA of sheep NMU (NCBI Reference Sequence: XM_027971397.1) has 645 bp (Supplementary file 1), and its coding region is 1–447 (Supplementary file l and 2). The translated protein has a total of 148 amino acids (Supplementary file 3). The transmembrane structure was predicted by TMHMM Server 2.0 software (https://services.healthtech.dtu.dk/service.php?TMHMM-2.0), and Editseq program of in DNAStar7.0 was used to intercept the extra-membrane part (1–447); then SignalP-5.0 Server software (https://services.healthtech.dtu.dk) /service.php?SignalP-5.0) was used to predict and cut off the signal peptide (1–37) of the NMU protein, and the number of corresponding base sequences of the remaining 111 amino acids were 336; then EditSeq program of DNAStar 7.0 was used to add the start codon ATG (methionine Met) and the Protean module. And then, the protein hydrophilicity and epitope of the 112 amino acid NMU were predicted (Supplementary file 4). Finally, the intercepted NMU bases were optimized, synthesized (Jinweizhi Biotechnology Co., Ltd.), connected with pET-28a( +) carrier (Solarbio Biotechnology Co., Ltd.), and transformed into DH5α competent cells (Solarbio Biotechnology Co., Ltd.), the extracted plasmid was sequenced and analyzed (Supplementary file 5), and the positive recombinant plasmid correctly sequenced was named pET-28a-NMU.

**Fig. 1:**
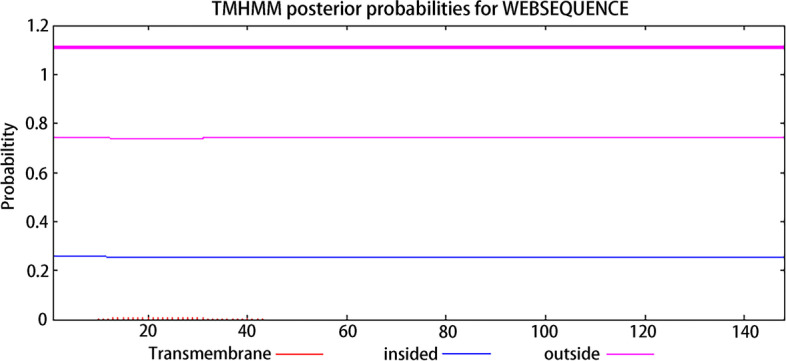
Prediction of sheep NMU protein transmembrane region

### Induced expression of pET-28a-NMU recombinant plasmid

The constructed pET-28a-NMU recombinant plasmid was transformed into the competent cell BL21 (ED3) (Solarbio Biotechnology Co., Ltd.). A single colony was picked and placed in sterilized LB liquid medium (containing Kan^+^), cultured at 37 ℃ until the OD_600_ value reached 0.6–0.8. The cells were collected after 6 h of induction with 1.0 mmol L^−1^ IPTG, freeze-thawed 3 times under -80 ℃, and sonicated until the liquid was clear (about 40 min) [44, 45]. The supernatant and precipitate were collected, respectively. Finally, all samples were detected by SDS–polyacrylamide gel electrophoresis (SDS-PAGE).

### Optimization of expression conditions for sheep NMU recombinant protein

In order to increase the expression level of NMU recombinant protein, the levels were detected by SDS-PAGE at different induction times (1 h, 2 h, 3 h, 4 h, 5 h, 6 h, 7 h, 8 h, 9 h). Under the optimal conditions, the NMU recombinant protein was highly expressed, the bacterial cells were disrupted by ultrasound, and the target protein was purified according to the procedures of His Tag Protein Purification Kit (Inclusion Body Protein) (CW0893S, CWBIO), and then detected by SDS-PAGE. The protein content was detected by UV spectrophotometer.

### Preparation of anti-sheep NMU polyclonal antibody

Two New Zealand white rabbits aged 8 weeks (male, 2.0 kg) were purchased from Experimental animal center of Lanzhou Veterinary Research Institute of Chinese Academy of Agricultural Sciences (CAAS). After the rabbits were adaptively fed for one week, the blood was collected from the ear veins and serum was separated as negative serum. The purified recombinant protein was mixed with the equal volume of Freund’s complete adjuvant, and after being fully emulsified, the rabbits were immunized by subcutaneous multi-point injection at back, scapular and popliteal lymph nodes at a dose of 800 μg per rabbit. One week later, the rabbits were immunized by subcutaneous injection at back and scapular at a dose of 400 μg per rabbit. After that, the immunization was boosted every 1 week (the method and dose were the same as the second time). Six days after the fourth immunization, the blood was collected by heart punctures to obtain rabbit anti-sheep NMU poly-antiserum.

### Serum titer detection of anti-sheep NMU polyclonal antibody

The purified NMU recombinant protein was used as the antigen, and 5 ug/well was used to coat the enzyme linked immunosorbent assay (ELISA) plate overnight at 4 °C. After washing, blocking and washing, the immune serum diluted from 1: 2000 to 1: 128,000 was added and incubated at 37 °C for 1 h. After washing, HRP-labeled goat anti-rabbit IgG (BOSTER Bioengineering Co., Ltd.) (diluted 1: 8000) was added and incubated at 37 ℃ for 1 h. After washing again, TMB substrate was added under dark conditions. After 15 min, 2 mol·L^−1^ H_2_SO_4_ was added to stop the reaction (50 μL/well). Finally, the absorbance values (OD) at 450 nm were detected with microplate reader. The highest dilution times of OD _test_ / OD _negative_ ≥ 2.1 is used as the titer of the poly-antiserum.

### Western blotting analysis of anti-sheep NMU polyclonal antibody

According to the operation steps of the whole protein extraction kit (strong) (BC3710, Solarbio), the total protein was extracted from the normal sheep small intestine. The purified NMU recombinant protein and the extracted total protein were electrophoresed on 12% SDS-PAGE, the SDS–polyacrylamide gel was cut at the 13 kDa, and then transferred to polyvinylidene fluoride (PVDF) membrane. Then the PVDF membrane was blocked with Tween-20 (TBS-T) containing 50 g/L skimmed milk powder for 2 h at 37 ℃ and incubated with rabbit antiserum (diluted 1: 500) overnight at 4 ℃. After washing 3 times with TBS-T, the PVDF membrane was incubated with HRP-labeled goat anti-rabbit IgG (diluted 1: 8000) for 2 h at room temperature. Finally, after washing 3 times with TBS-T, ECL luminescent solution was dropwise added for color development.

### Indirect immunofluorescence staining for NMU

The paraffin sections of the formaldehyde-fixed samples were made by conventional methods, and the indirect immunofluorescence staining was performed as follows: after deparaffination, sections were placed into a repair box filled with EDTA buffer (pH 8.0) for antigen retrieval in a microwave oven (medium heat for 8 min to boiling, stop fire for 8 min, and then change into medium–low heat for 7 min; the buffer should be prevented from over-evaporating during this process, and the slides should not be dried). After natural cooling, the sections were placed into PBS (pH 7.4) and washed for 5 min × 3 times. After shaking off the excess liquid, a circle was draw around the tissue with a histochemical pen to prevent the antibody from flowing away, the PBS was dried, 10% donkey serum was added to block for 30 min; after gently shaking off the blocking reagent, the diluted primary antibody was added to the sections, which were laid flat in a humid box at 4 °C and incubated overnight. Then the sections were put into PBS (pH 7.4) and washed for 5 min × 3 times. After being dried slightly, the secondary antibody (Goat Anti-Rabbit IgG H&L (Alexa Fluor® 488) ab150077, abcam) was added into the circle to cover the tissue, the sections were incubated at room temperature in the dark for 50 min; then the liquid was shaken off and the sections were washed in PBS (pH 7.4) for 5 min × 3 times. After being dried slightly, DAPI staining solution was added and the sections were incubated for 10 min at room temperature in the dark, and then washed in PBS (pH 7.4) for 5 min × 3 times. The autofluorescence quencher were added to the circle to incubate for 5 min and rinsed with running water for 10 min. After the sections were dried slightly, they were mounted with anti-fluorescence quenching mounting medium. The distribution characteristics of NMU in the sheep small intestine were observed and the images were collected by the fluorescence microscope (The DV Elite™ Imaging System, GE, USA). DAPI ultraviolet excitation wavelength was 330–380 nm, emission wavelength was 420 nm, emitting blue light; FITC excitation wavelength was 465–495 nm, emission wavelength was 515–555 nm, emitting green light.

### Detection of NMU expression level

The frozen tissue samples were thawed on ice. The 1.0 g of tissue was weighed accurately, 1 ml of PBS and two magnetic beads were added, which was homogenized at -10 °C for 15 min, and centrifuged for 10 min (4 °C, 12,000 rpm). The supernatant was collected. The protein concentration was measured by BCA protein Assay Kit (BCA protein Assay Kit, Cat#PC0020, Lot. No, 20,210,908, Solarbio, Beijing, China), and the NMU expression level in each segment of sheep intestine was detected by ELISA (Sheep NMU ELISA Kit, Shanghai Enzyme Linked Biotechnology Co., LTD). The inter- and intra-assay coefficients of variability for this ELISA KIT assay were less than 10% and 15%, respectively, the test results are considered reliable.

### Statistical analysis

All data were presented as mean ± standard deviation (SD). One-way analysis of variance (ANOVA) with Duncan’s multiple range test was used to analyze the differences among various parts in the same group; the independent T test was used to analyze the differences between the same parts of the infected group and the control group, using SPSS 23.0 (SPSS Inc., Chicago, USA), the difference was statistically significant at *P* < 0.05.

All methods were carried out in accordance with relevant guidelines and regulations.

## Results

### Sheep NMU gene synthesis and recombinant plasmid construction

**Fig. 2:**
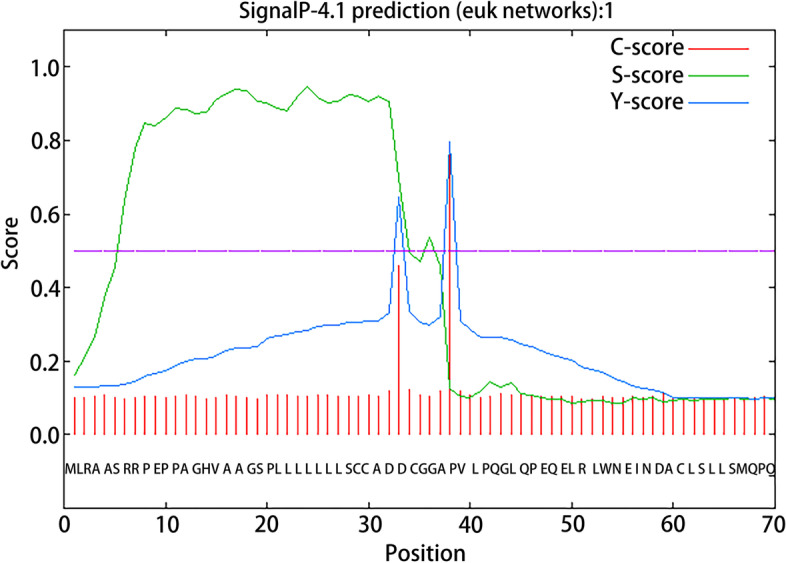
Prediction of sheep NMU protein signal peptide

**Fig. 3:**
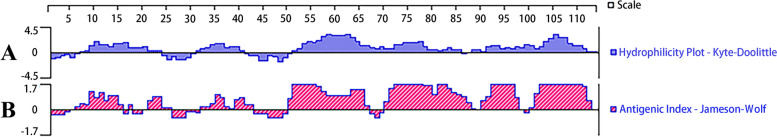
Prediction of the hydrophilicity and epitope of sheep NMU

The transmembrane structure of NMU was predicted, and a total of 148 amino acids were all extra-membrane regions (Fig. 1). The extra-membrane part was cut by Editseq program of DNAStar7.0; the signal peptide prediction results showed that amino acids 1–37 of the protein were the signal peptide part (Fig. 2), and which was cut off. There were 111 amino acids left, and then the start codon ATG was added upstream of the protein, and finally the hydrophilicity (Fig. 3A), epitope (Fig. 3B) and molecular weight of NMU recombinant protein were predicted. The results showed that the protein was a hydrophobic protein with a high antigenic index and the molecular weight was 13 kDa.

### Induced expression results of pET-28a-NMU recombinant plasmid

**Fig. 4:**
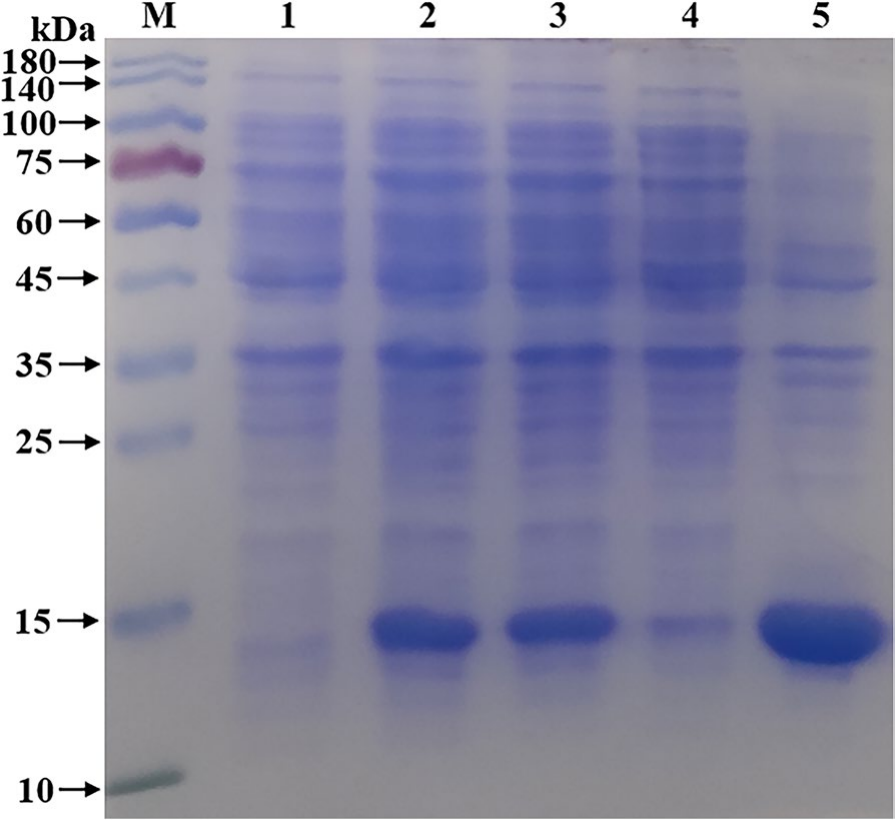
SDS-PAGE detection of NMU recombinant protein expression**.** **M** Protein molecular weight Marker; **1** Recombinant bacteria pre-induction products; **2–3** Recombinant bacteria induced products; **4** Supernatant of recombinant bacteria induced products; **5** Precipitation of recombinant bacteria induced products

The recombinant bacteria were cultured at 37 ℃ until the OD_600_ value was 0.8. After 6 h of induction with IPTG (1.0 mmol/L), the bacteria were collected. After sonication, the supernatant and precipitate were collected. Compared with the products without induction by recombinant bacteria, the post-induction products appeared obvious expression bands (Fig. 4); after ultrasonic disruption and centrifugation, the target bands only appeared in the precipitation of recombinant bacteria-induced products, indicating that the recombinant protein NMU mainly in the form of inclusion body was successfully expressed in BL21, and the size was 13 kDa, which was congruent with the expectations.

### Optimization results of expression conditions for sheep NMU recombinant protein

**Fig. 5:**
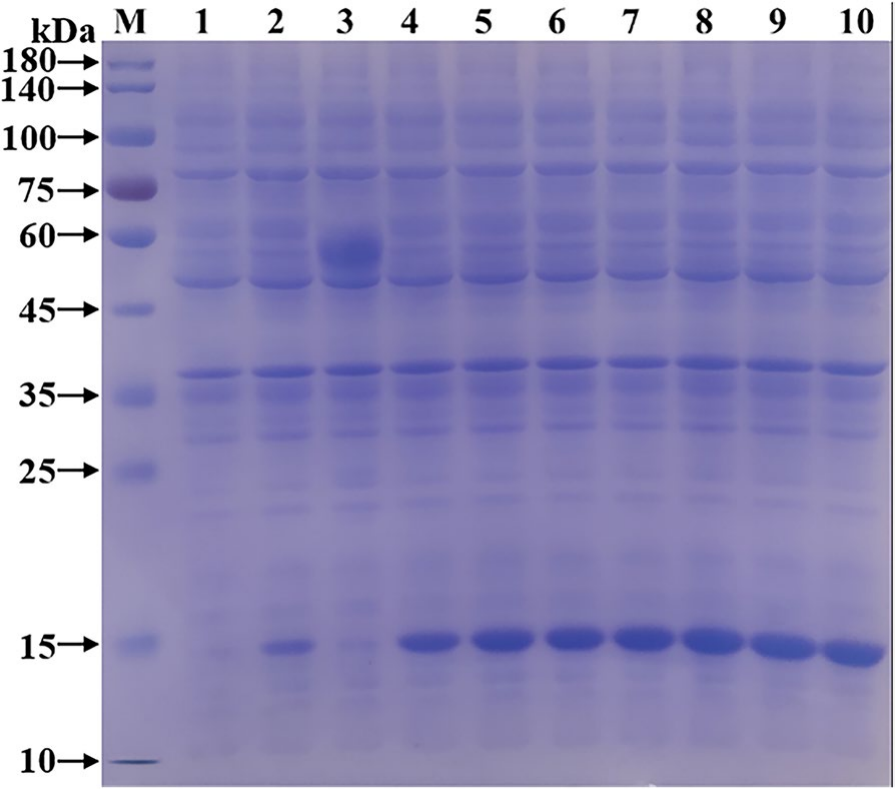
The optimization of expression time of sheep NMU recombinant protein. **M** Protein molecular weight Marker; **1** Recombinant bacteria pre-induction products; **2–10** Products of recombinant bacteria induced for 1 h, 2 h, 3 h, 4 h, 5 h, 6 h, 7 h, 8 h, 9 h

The recombinant bacteria were induced to express NMU at different times (1–9 h). The target protein was expressed when IPTG was added for 1 h by SDS-PAGE detection. As time passed, the expression level gradually increased, and reached the highest level at 4 h (Fig. 5). Therefore, the optimal induction time for NMU recombinant protein expression was at 4 h.

### Purification results of sheep NMU recombinant protein

**Fig. 6:**
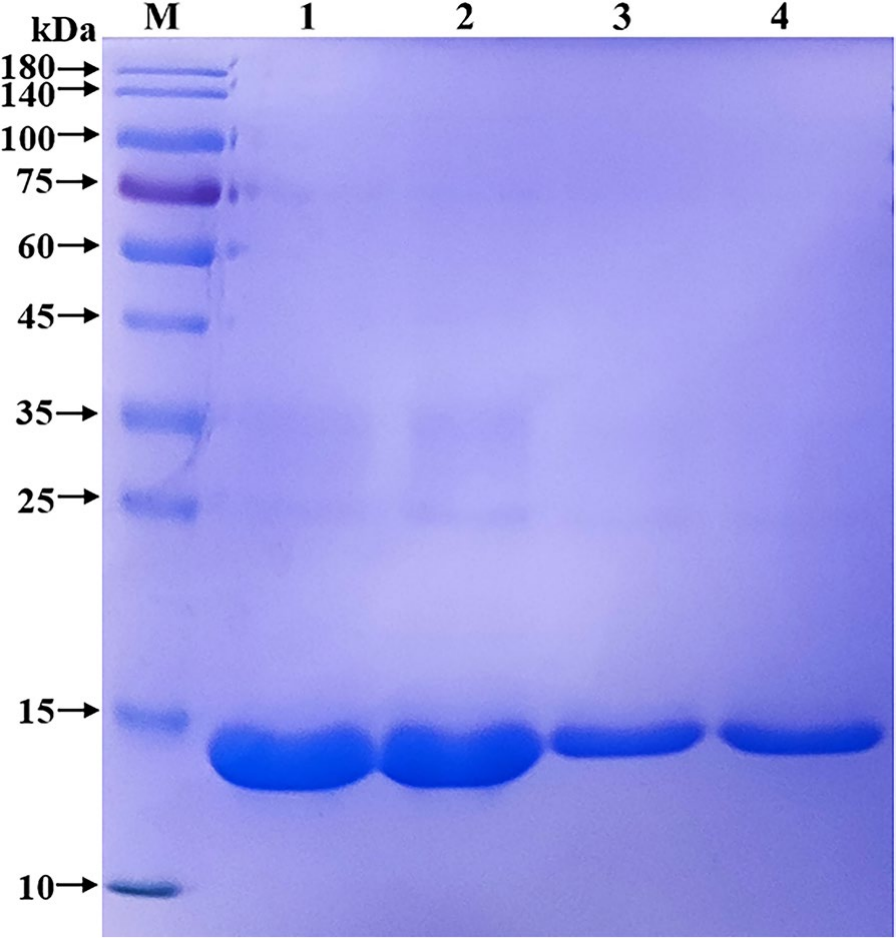
Purification of sheep NMU recombinant protein**. M** Protein molecular weight Marker; **1–4** Purified recombinant protein

The NMU recombinant protein was expressed under optimal expression conditions, and the eluate was collected during purification. The maximum protein content determined by UV spectrophotometer was 2.544 mg⋅mL^−1^. The purity of the purified recombinant protein detected by SDS-PAGE was high, and the protein size was about 13 kDa (Fig. 6).

### Serum titer and specificity detection of anti-sheep NMU polyclonal antibody

The antibody titer was determined by indirect ELISA. When the antiserum and the pre-immune negative serum were diluted from 1: 32,000 and 1: 2000, respectively, OD_450_ (positive)/OD_450_ (negative) > 2.1, indicating that the antibody serum titer is 1: 32,000.

**Fig. 7:**
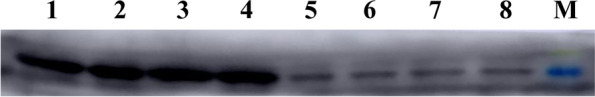
Western blotting results of polyclonal antibody against NMU.** 1–4** Purified recombinant protein; **5–8** Total natural protein extracted; **M** Protein molecular weight Marker, 13 kDa, the SDS–polyacrylamide gel was cut at the 13 kDa, and then transferred to polyvinylidene fluoride (PVDF) membrane. The result was only provided the closely cropped and no full-length image (Supplementary file 6)

The specificity results showed that there was a clear western blot at about 13 kDa on the PVDF membrane (Fig. 7, Supplementary file 6), indicating that the rabbit anti-sheep NMU antibody could specifically bind to recombinant proteins and natural proteins, and it had reactogenicity.

### Impact of *Moniezia benedeni* infection on NMU expression in sheep small intestine

**Fig. 8:**
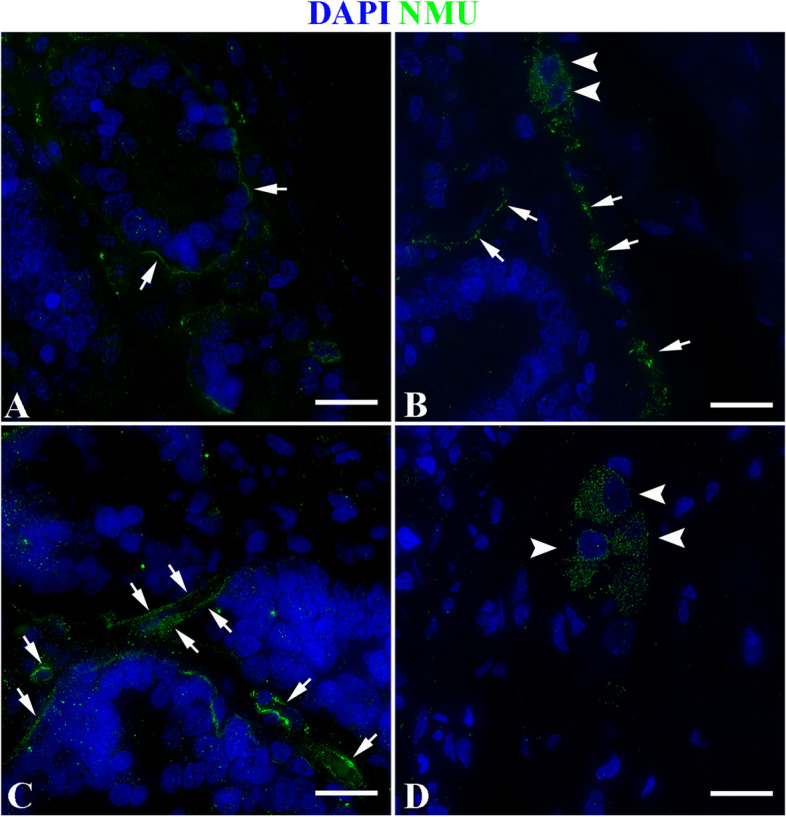
NMU distribution in duodenum. **A** The expression characteristics of NMU on the nerve fibers at the base of the intestinal gland in the control group; **B** The expression characteristics of NUM in nerve cell bodies and fibers in the submucosa of the control group; **C** The NMU expression characteristics in the nerve fibers at the base of the gland in the infected group. **D** The NMU expression characteristics in nerve cell bodies in the submucosa of the infection group. The white arrow indicates the nerve fiber, and the white triangle arrow indicates the nerve fiber cell body; Scale bar is 50 μm

**Fig. 9:**
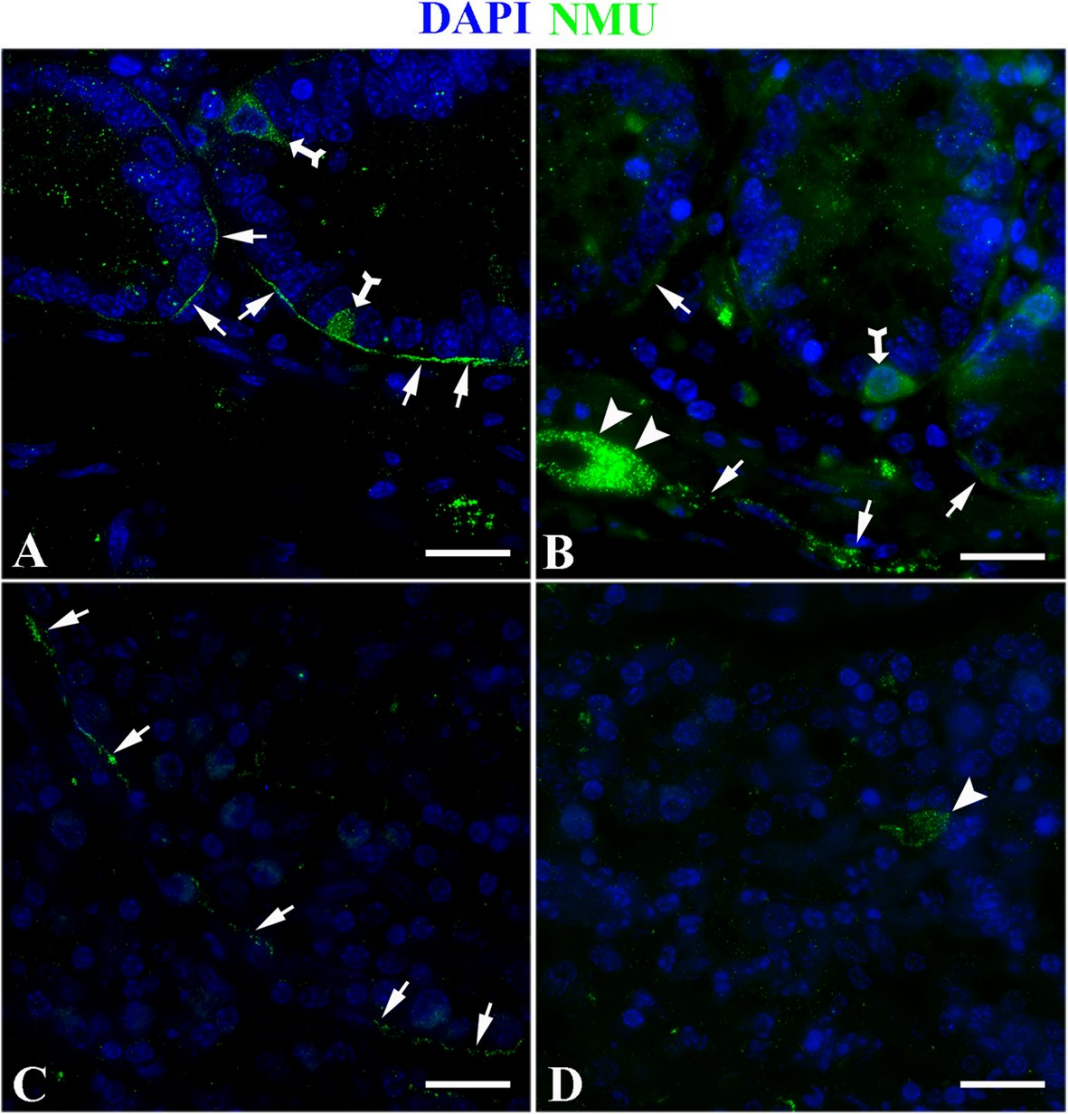
NMU distribution in the jejunum. **A** The expression characteristics of NMU in the nerve fibers of the intestinal glands in the control group; **B** The expression characteristics of NUM in nerve cell bodies and fibers in the submucosa in the control group; **C** The expression characteristics of NMU in the nerve fibers of the glands in the infected group; **D** The expression characteristics of NMU in nerve cell bodies in the submucosa in the infected group. The white arrow shows the nerve fiber, the white triangle arrow shows the nerve fiber cell bodies; the bifurcated arrow shows the cells that specifically express NMU in the glandular epithelium; Scale bar is 50 μm

**Fig. 10:**
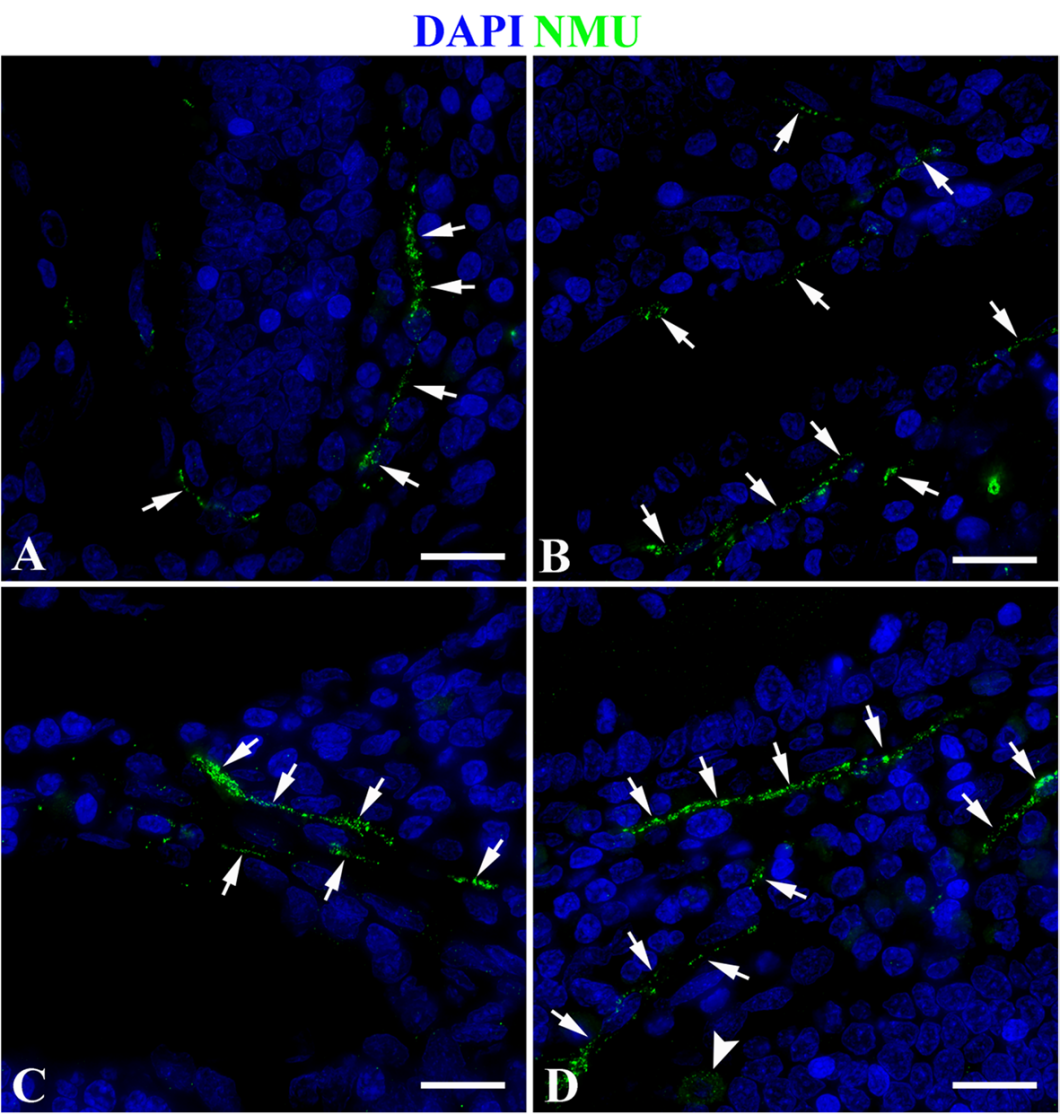
Distribution of NMU in the ileum. **A** The NMU expression characteristics on nerve fibers at the base of the intestinal glands in the control group; **B** The expression characteristics of NMU in nerve cell bodies and fibers in the submucosa of the control group; **C** The expression characteristics of NMU in nerve fibers at the base of the glands in the infected group; **D** The expression characteristics of NMU in nerve fiber in the submucosa of the infection group. The white arrow indicates the nerve fiber, and the white triangle arrow indicates the nerve fiber cell body; Scale bar is 50 μm

The results showed that in the duodenum of the control group, NMU was mainly expressed specifically on the nerve fibers around the subepithelial *lamina propria* (LP) of the intestinal glands (Fig. 8A), and specific expression was also seen on the ganglion cell bodies and fibers in the submucosa (Fig. 8B). In the duodenum of the infected group, the location of nerve fibers expressing NMU was similar to that of the control group (Fig. 8C-D). In the jejunum of the control group, in addition to the nerve fibers in the LP around the intestinal glands and the ganglia of the submucosa, NMU also specifically expresesed in some cells scattered in the epithelium of the intestinal glands (Fig. 9A-B); in the jejunum of the infected group, the location of nerve fibers expressing NMU was similar to that of the control group (Fig. 9C-D). In the ileum of the control group, the expression characteristics of NMU were completely similar to those of the duodenum. It was also mainly expressed in the nerve fibers of the LP around the intestinal glands and the ganglia of the submucosa (Fig. 10A-B). The ileum of the infected group and the control group have similar expression characteristics (Fig. 10C-D).

**Fig. 11:**
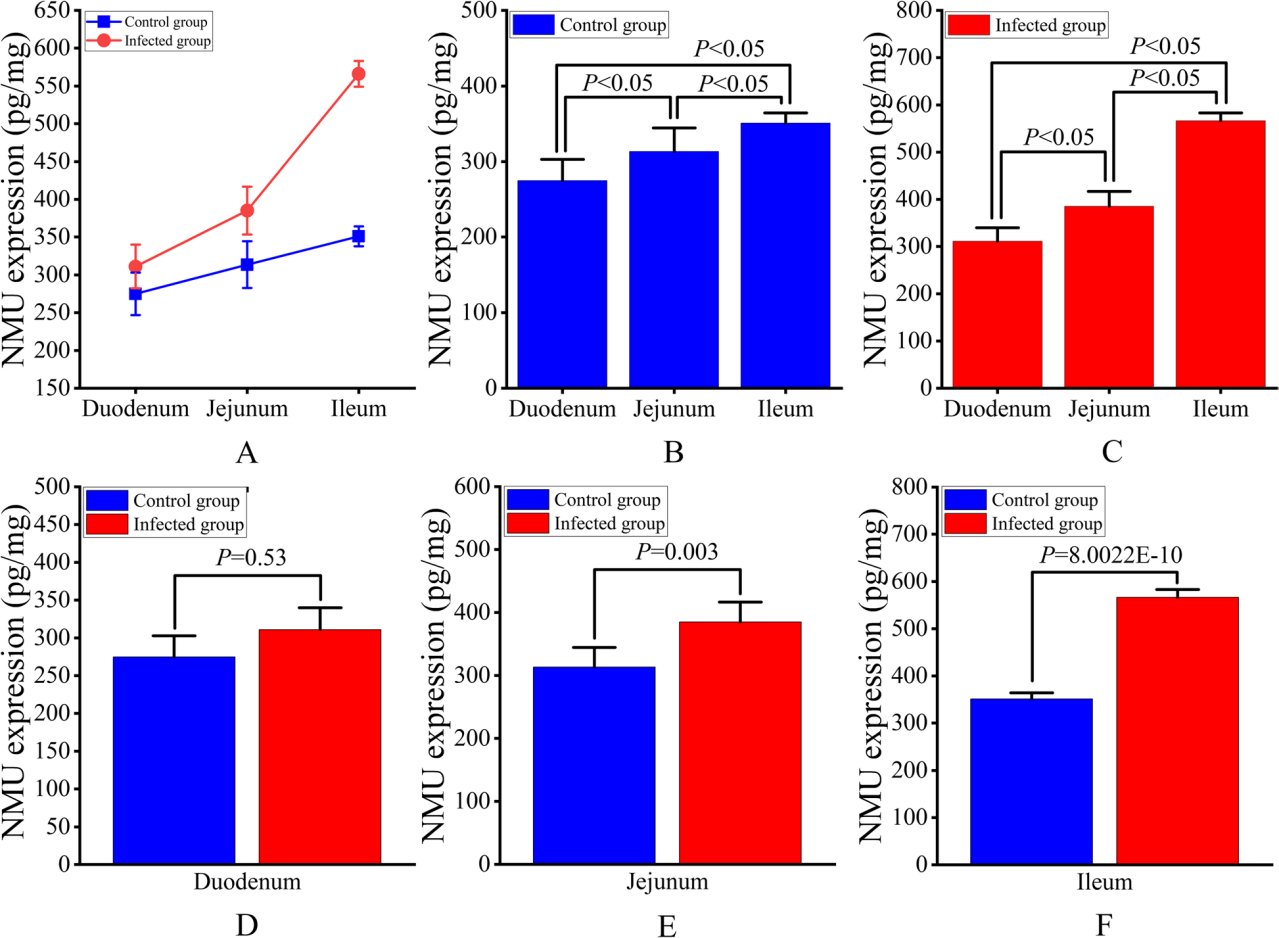
NMU expression levels in the small intestine of sheep

ELISA test results showed that the expression of sheep NMU gradually increased from duodenum, jejunum to ileum (Fig. 11A), and differed significantly from each other (*P* < 0.05) (Fig. 11B). The change trends of NMU expression from duodenum to ileum in infected group was similar to that in the control group (Fig. 11A), but the expression levels among duodenum, jejunum and ileum differed significantly from each other (*P* < 0.05) (Fig. 11C). In particular, the expression of NMU in the jejunum and ileum in the infected group was significantly higher than that in the control group (*P* < 0.05) (Fig. 11D-F).

## Discussion

Immunofluorescence results showed that the nerve fibers that specifically express NMU in the small intestine of normal sheep mainly extend from the ganglion in the submucosal LP to the LP, and the expression level was relatively high; especially high in nerve fibers in the LP around the intestinal glands. From duodenum, jejunum to ileum, the fluorescence signal gradually enhanced, and the expression level in jejunum and ileum was significantly higher than that in duodenum. Some studies have shown that in addition to the wide expression of NMU in the central nervous system, the intestine was one of its high expression sites in the periphery. For instance, it could be expressed in the intestine of humans [46], pigs [47, 48], guinea pigs [49], rat [50]. In the rat intestine, NMU was mainly expressed in the rat’s intestinal mucosa and nerve cell bodies and fibers in the intestinal muscular layer [50], and the highest expression was in the duodenum and jejunum [50], the above researches were almost consistent with the results in the present study. This provides a morphological basis for NMU extensively participating in the regulation of intestinal immune homeostasis in sheep small intestine.

Further analysis showed that *Moniezia benedeni* infection did not change the spatial distribution of nerve fibers expressing NMU, but the infection resulted in an increase of NMU expression in each intestinal segment. Apart from duodenum (*P* > 0.05) it was significantly increased in the jejunum and ileum (*P* < 0.05). Veterinary anatomicpathological results showed that the scolex of *Moniezia benedeni* was mainly located in the anterior part of the small intestine, such as in the duodenum, and then extended backward along the intestinal lumen, especially the pregnant proglottides, were mainly located in the jejunum and ileum, etc. However, studies suggested that, on proglottides of *Moniezia benedeni* there are great differences in the organizational structure, nutrient absorption, and metabolism during different developmental stages period [51]. Recent studies have shown that cholinergic neurons expressing NMU can directly perceive the intestinal alarmin IL-33 and parasite metabolites, and effectively promote the expression of NMU, which is activated by the MYD88 pathway through the Toll-like receptor [17, 43, 52]. It is now known that NMU exerts the specific biological functions depending on its two specific receptors NMUR1 and NMUR2, which both have 7 transmembrane domains and are typical G-protein coupled receptor (GPCR). Peripheral organs mainly expressed NMUR1 [41]. The latest studies demonstrated that NMUR1 was also expressed on the ILC2 cell membrane in the LP of the small intestine, and the NMU expression was significantly increased when there was parasite infection, and NMUR1 could effectively activate ILC2, in turn, initiate anti-parasitic infection immunity [17, 42, 43]. Hence, we speculate that, in different intestinal segments, the differences of NMU levels were closely related to the development, stimulation and metabolism of proglottides. Above all, it was suggested that the *Moniezia benedeni* infection could stimulate the sheep intestine to significantly highly express NMU, and then trigger the antiparasitic immune response with activation of ILC2 as the core. It was one of the effective potential mechanisms of the mucosal immunity of the digestive tract of sheep against the *Moniezia bechii* infection.

In addition, our results also showed that the levels of NMU expression were different in sheep intestinal segments. Studies have demonstrated that NMU secreted by enteric nerves has multiple functions [53–55]. However, their microenvironments and functions are different in different segments, such as the colonization flora, the composition of the mucosal epithelium, and local immunity [55]. Therefore, in healthy sheep intestine, the differences of NMU expression levels in each segment is a display for their physiological differences. In the intestinal gland epithelium of jejunum, scattered NMU positive cells were co-localized with NMU nerve fibers. Judging only by morphology, it seems to be closer to intestinal pluripotent stem cells or endocrine cells. While the pathological study proved that the infection of *Moniezia bechii* leads to the excessive proliferation of mucosal epithelium, intestinal gland epithelium, goblet cells and Paneth cells and hypersecretion of mucus. Researches on NMU also showed that NMU coexisted with calcitonin gene-related peptide (CGRP), substance P (SP) [47, 48] or vasoactive intestinal peptide (VIP) and immunoactive neurons of neuropeptide Y (NPY) [49]. Therefore, NMU in the intestine may have a potential connection with the differentiation of the mucosal epithelial cells and the secretion of mucus and antimicrobial peptides, etc., suggesting that NMU plays a more complex function in the intestine, but it needs further research to confirm.

## Conclusions

In the present study, the sheep NMU prokaryotic recombinant protein and a specific rabbit anti-sheep NMU polyclonal antibody, with a molecular weight of about 13 kDa, were successfully prepared. The nerve fibers specifically expressing NMU mainly extend from the ganglion in the submucosal LP to the LP in the sheep small intestine, and the expression level was relatively high; especially on the nerve fibers of LP around the intestinal glands. Its expression level gradually increases from the duodenum to the ileum. In addition, scattered NMU positive cells were distributed in the epithelium of the jejunal crypts. *Moniezia benedeni* infection did not change the spatial distribution of NMU-expressing nerve fibers, but induced the expression level was increased in each intestinal segment. It was suggested that *Moniezia benedeni* infection could stimulate the NMU high expression in the sheep enteric nervous, and which provided a basis for further studies on whether NMU exerts anti-parasitic immunity by activating ILC2. In addition, the NMU expressed in some intestinal gland epitheliums also provided a basis for studying its roles in cell differentiation, mucus and antimicrobial peptide secretion in the intestinal mucosal epithelium. The present study would lay the foundation for further revealing the molecular mechanism of sheep’s neural-immune interaction network to perceive the colacobiosis of parasites.

## Supplementary Information

**Additional file 1.**

**Additional file 2.**

**Additional file 3.**

**Additional file 4.**

**Additional file 5.**

**Additional file 6.**

## Data Availability

All of the data generated or analyzed during this study are available from the corresponding author on reasonable request.

## Abbreviations

NMU: Neuromedin U
ILC2s: Group 2 innate lymphoid cells
ENS: Enteric nervous system
BMP-2: Bone morphogenetic protein 2
ILC3s: Group 3 innate lymphoid cells
IL: Interleukin
ELISA: Enzyme linked immunosorbent assay
GPCR: G-protein coupled receptor
CGRP: Calcitonin gene-related peptide
SP: Substance P
VIP: Vasoactive intestinal peptide
NPY: Neurons of neuropeptide Y
LP: Lamina propria
