# Supplementary material for: Moniezia benedeni infection enhances neuromedin U (NMU) expression in sheep (Ovis aries) small intestine
Source: BMC Vet Res. 2022 Apr 19;18:143. doi: 10.1186/s12917-022-03243-2 (PMC9016964; doi:10.1186/s12917-022-03243-2)
Supplement: Supplementary file 2 — Additional file 2. [file 12917_2022_3243_MOESM2_ESM.docx]

**PREDICTED: Ovis aries neuromedin U (NMU), mRNA**

**NCBI Reference Sequence: XM_027971397.1**

**CDs 1-447**

1 atgctgcgag ccgcgagccg tcgccccgag ccgccggcgg ggcatgtggc cgccgggtcc

61 ccgctcctgc tgctgctact gctgtcttgt tgcgcggacg actgcggagg tgctccagta

121 ttgcctcaag gattacagcc tgaacaagaa ttacggttgt ggaatgagat aaatgatgct

181 tgtttgtctt tgttatccat gcagccacag cctcaggcat ccaatgcatt ggaggagatt

241 tgcctcacaa ttatgcggac tctaccaaag ccccaggaaa cagatgaaaa agataacacc

301 aaaaggttct tatttcatta ttcgaagact cgaaagttgg gcaattcaaa tgttgtggaa

361 gaattccaag gtcctattgc aagccaaagt agaagatact ttttattcag gccacgcaat

421 ggaagaagat cagaaggtta catttaa
