# Supplementary material for: Moniezia benedeni infection enhances neuromedin U (NMU) expression in sheep (Ovis aries) small intestine
Source: BMC Vet Res. 2022 Apr 19;18:143. doi: 10.1186/s12917-022-03243-2 (PMC9016964; doi:10.1186/s12917-022-03243-2)
Supplement: Supplementary file 3 — Additional file 3. [file 12917_2022_3243_MOESM3_ESM.docx]

**NCBI Reference Sequence: XM_027971397.1**

**CDS 1..447**

**Translated into protein, a total of 148 amino acids as follows:**

/translation="MLRAASRRPEPPAGHVAAGSPLLLLLLLSCCADDCGGAPVLPQGLQPEQELRLWNEINDACLSLLSMQPQPQASNALEEICLTIMRTLPKPQETDEKDNTKRFLFHYSKTRKLGNSNVVEEFQGPIASQSRRYFLFRPRNGRRSEGYI"
