# Supplementary material for: Moniezia benedeni infection enhances neuromedin U (NMU) expression in sheep (Ovis aries) small intestine
Source: BMC Vet Res. 2022 Apr 19;18:143. doi: 10.1186/s12917-022-03243-2 (PMC9016964; doi:10.1186/s12917-022-03243-2)
Supplement: Supplementary file 4 — Additional file 4. [file 12917_2022_3243_MOESM4_ESM.docx]

**The signal peptide is predicted for the 148 amino acid sequence, 1-37 is the signal peptide part, the signal peptide is truncated, and the remaining 111 amino acids are as follows:**

APVLPQGLQPEQELRLWNEINDACLSLLSMQPQPQASNALEEICLTIMRTLPKPQETDEKDNTKRFLFHYSKTRKLGNSNVVEEFQGPIASQSRRYFLFRPRNGRRSEGYI.

**The corresponding base sequence is as follows:**

gctccagtattgcctcaaggattacagcctgaacaagaattacggttgtggaatgagataaatgatgcttgtttgtcttt

gttatccatgcagccacagcctcaggcatccaatgcattggaggagatttgcctcacaattatgcggactctaccaaagc

cccaggaaacagatgaaaaagataacaccaaaaggttcttatttcattattcgaagactcgaaagttgggcaattcaaat

gttgtggaagaattccaaggtcctattgcaagccaaagtagaagatactttttattcaggccacgcaatggaagaagatc

agaaggttacatttaa

**Add the starting amino acid M, a total of 112 amino acids, 13kD, the results are as follows:**

MAPVLPQGLQPEQELRLWNEINDACLSLLSMQPQPQASNALEEICLTIMRTLPKPQETDEKDNTKRFLFHYSKTRKLGNSNVVEEFQGPIASQSRRYFLFRPRNGRRSEGYI.

**Add the start codon atg, a total of 339 bases（Including stop codon taa）, and the results are as follows:**

atggctccagtattgcctcaaggattacagcctgaacaagaattacggttgtggaatgagataaatgatgcttgtttgtc

tttgttatccatgcagccacagcctcaggcatccaatgcattggaggagatttgcctcacaattatgcggactctaccaa

agccccaggaaacagatgaaaaagataacaccaaaaggttcttatttcattattcgaagactcgaaagttgggcaattca

aatgttgtggaagaattccaaggtcctattgcaagccaaagtagaagatactttttattcaggccacgcaatggaagaag

atcagaaggttacatttaa
