# Supplementary material for: Moniezia benedeni infection enhances neuromedin U (NMU) expression in sheep (Ovis aries) small intestine
Source: BMC Vet Res. 2022 Apr 19;18:143. doi: 10.1186/s12917-022-03243-2 (PMC9016964; doi:10.1186/s12917-022-03243-2)

**Gene sequence information of the sheep (Ovis aries) NMU**

BamHI and XhoI are absent in the optimized sequence NMU

**1. The original sequence of NMU**

GGATCCATGGCTCCAGTATTGCCTCAAGGATTACAGCCTGAACAAGAATTACGGTTGTGGAATGAGATAAATGATGCTTGTTTGTCTTTGTTATCCATGCAGCCACAGCCTCAGGCATCCAATGCATTGGAGGAGATTTGCCTCACAATTATGCGGACTCTACCAAAGCCCCAGGAAACAGATGAAAAAGATAACACCAAAAGGTTCTTATTTCATTATTCGAAGACTCGAAAGTTGGGCAATTCAAATGTTGTGGAAGAATTCCAAGGTCCTATTGCAAGCCAAAGTAGAAGATACTTTTTATTCAGGCCACGCAATGGAAGAAGATCAGAAGGTTACATTTAACTCGAG

**2. The Optimized (for *Escherichia coli(E.coli)*) sequence of NMU**

A 95 T 61 C 101 G 94 | GC%: 55.56% | Length: 351

GGATCCATGGCGCCGGTGCTGCCGCAAGGCCTGCAGCCGGAACAAGAACTGCGCCTGTGGAACGAAATTAACGATGCGTGCCTGAGCCTGCTGAGCATGCAGCCGCAGCCGCAAGCGAGCAACGCGCTGGAAGAAATTTGCCTGACCATTATGCGCACCCTGCCGAAACCGCAAGAAACCGATGAAAAAGATAACACCAAACGCTTTCTGTTTCATTATAGCAAAACCCGCAAACTGGGCAACAGCAACGTGGTGGAAGAATTTCAAGGCCCGATTGCGAGTCAGAGCCGCCGCTATTTTCTGTTTCGCCCGCGCAACGGCCGCCGCAGCGAAGGCTATATTTAACTCGAG

**GC Content**


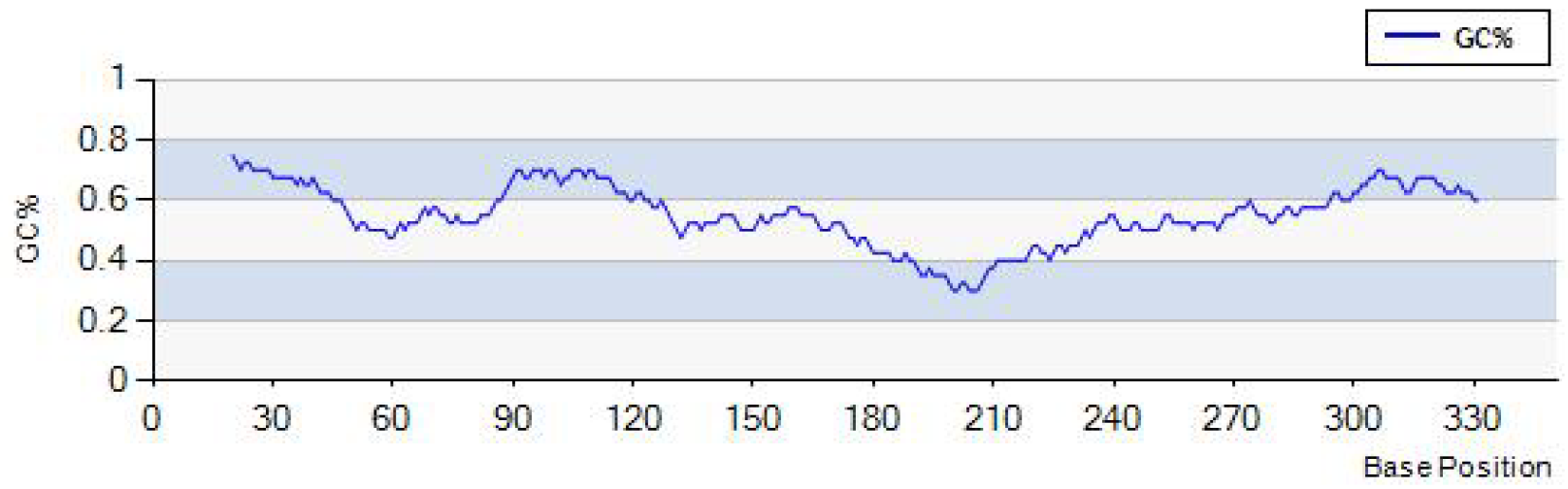

Supplement: Supplementary file 5 — Additional file 5. [file 12917_2022_3243_MOESM5_ESM.docx]
