# Supplementary material for: Moniezia benedeni infection enhances neuromedin U (NMU) expression in sheep (Ovis aries) small intestine
Source: BMC Vet Res. 2022 Apr 19;18:143. doi: 10.1186/s12917-022-03243-2 (PMC9016964; doi:10.1186/s12917-022-03243-2)
Supplement: Supplementary file 6 — Additional file 6. [file 12917_2022_3243_MOESM6_ESM.docx]

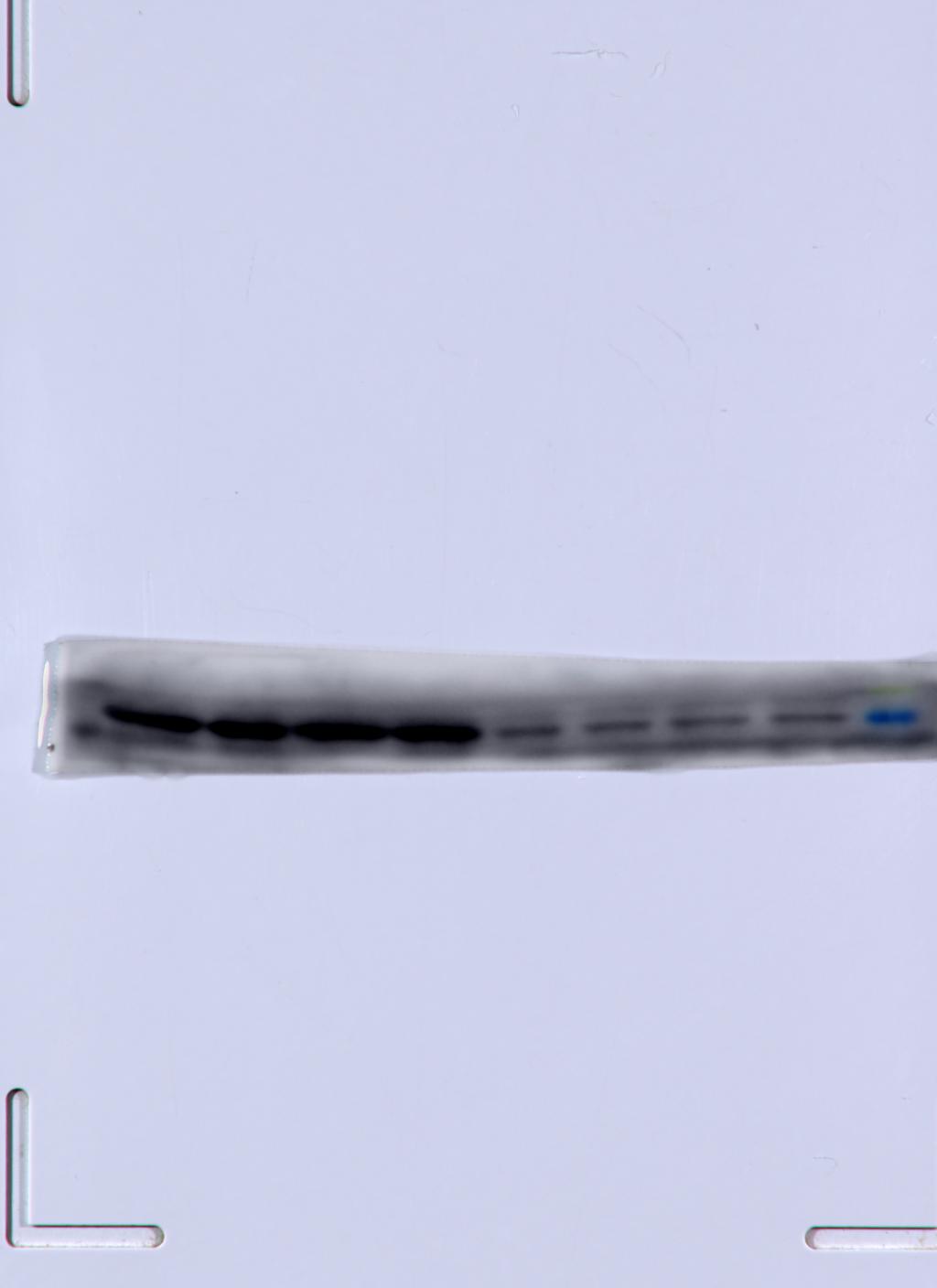


**Supplementary file 6** The original image of Western blotting results of polyclonal antibody against NMU.
